# Inhibition of NAADP signalling on reperfusion protects the heart by preventing lethal calcium oscillations via two-pore channel 1 and opening of the mitochondrial permeability transition pore

**DOI:** 10.1093/cvr/cvv226

**Published:** 2015-09-22

**Authors:** Sean M. Davidson, Kirsty Foote, Suma Kunuthur, Raj Gosain, Noah Tan, Richard Tyser, Yong Juan Zhao, Richard Graeff, A. Ganesan, Michael R. Duchen, Sandip Patel, Derek M. Yellon

**Affiliations:** 1 The Hatter Cardiovascular Institute, University College London, 67 Chenies Mews, WC1E 6HX London, UK; 2 School of Chemistry, University of Southampton, Highfield, Southampton, UK; 3 Department of Physiology, Li Ka Shing School of Medicine, The University of Hong Kong, Hong Kong, China; 4 School of Pharmacy, University of East Anglia, Norwich, UK; 5 Department of Cell and Developmental Biology, University College London, London, UK

**Keywords:** NAADP, Ischaemia, Reperfusion, Calcium, Lysosomes

## Abstract

**Aims:**

In the heart, a period of ischaemia followed by reperfusion evokes powerful cytosolic Ca^2+^ oscillations that can cause lethal cell injury. These signals represent attractive cardioprotective targets, but the underlying mechanisms of genesis are ill-defined. Here, we investigated the role of the second messenger nicotinic acid adenine dinucleotide phosphate (NAADP), which is known in several cell types to induce Ca^2+^ oscillations that initiate from acidic stores such as lysosomes, likely via two-pore channels (TPCs, TPC1 and 2).

**Methods and results:**

An NAADP antagonist called Ned-K was developed by rational design based on a previously existing scaffold. Ned-K suppressed Ca^2+^ oscillations and dramatically protected cardiomyocytes from cell death *in vitro* after ischaemia and reoxygenation, preventing opening of the mitochondrial permeability transition pore. Ned-K profoundly decreased infarct size in mice *in vivo*. Transgenic mice lacking the endo-lysosomal TPC1 were also protected from injury.

**Conclusion:**

NAADP signalling plays a major role in reperfusion-induced cell death and represents a potent pathway for protection against reperfusion injury.

## 1. Introduction

An interruption in blood supply to the myocardium, such as occurs during myocardial infarction or during cardiopulmonary bypass, leads to ischaemia which can cause irreversible injury. Restoration of blood supply is essential to salvage the myocardium; however, this paradoxically results in further injury to the myocardium.^1^ Ischaemia and reperfusion cause major changes in intracellular ATP levels, redox state, pH, [Ca^2+^], and oxidative stress—critical factors leading to opening of the mitochondrial permeability transition pore (mPTP).^2–4^ This non-selective pore on the inner mitochondrial membrane results in mitochondrial depolarization and swelling, and inevitably necrotic cell death.^2,4^ Chemical inhibition or genetic ablation of cyclophilin D (cypD), a protein known to regulate mPTP opening, significantly increases the resistance of the heart to ischaemia and reperfusion injury.^5,6^

Despite its role in cell death, Ca^2+^ is an essential ion in the heart, coupling electrical excitation to muscular contraction. During the cardiac action potential, Ca^2+^ influx via the sarcolemmal L-type Ca^2+^ channels triggers Ca^2+^ release from the sarcoplasmic reticulum (SR) via ryanodine receptors (RyRs). The resulting elevation of [Ca^2+^]_c_ stimulates cardiomyocyte contraction. In healthy cardiomyocytes, therefore, [Ca^2+^]_c_ is very finely regulated via homeostatic mechanisms, principally by buffering within the SR but also in other organelles such as mitochondria and lysosomes.^7,8^ When ATP becomes depleted during ischaemia, however, [Ca^2+^]_c_ increases.^9–11^ Reperfusion is necessary to save the cells, but during the first few minutes of reperfusion, Ca^2+^ is repeatedly released from the SR and pumped back in a futile oscillatory cycle. If normal Ca^2+^ levels are not quickly restored, this can result in hypercontracture and cell death.^7,12,13^ The Ca^2+^ oscillations are caused by cycles of SR Ca^2+^ release and re-uptake, leading to mitochondrial Ca^2+^ overload and mPTP opening. Consequently, drugs such as ryanodine that inhibit the RyR prevent these oscillations, and protect against mPTP opening and cell death.^12,14^ Studies in cardiomyocytes have led to the proposal that the SR–mitochondrial interaction is a critical target of reperfusion injury.^7,15^ Using multiphoton microscopy of the intact heart, we have recently shown that slow [Ca^2+^]_c_ waves occur in cardiomyocytes during reperfusion, and that these precede mPTP opening.^3^

Nicotinic acid adenine dinucleotide phosphate (NAADP) is the most potent Ca^2+^-mobilizing second messenger identified to date.^16^ Much evidence suggests that NAADP triggers Ca^2+^ release from acidic organelles such as lysosomes.^8,16–22^ It is therefore distinct from the other known second messengers, inositol triphosphate (IP_3_, which stimulates Ca^2+^ release from the ER/SR via IP_3_ receptors), and cyclic ADP ribose (which stimulates Ca^2+^ release from the SR via the RyR). The Ca^2+^-mobilizing properties of NAADP were initially identified in sea urchin eggs.^23^ Subsequent data have shown that NAADP can stimulate Ca^2+^ oscillations in diverse mammalian cell types, including pancreatic beta cells, smooth muscle, and endothelium.^20,24–26^ Two-pore channels (TPC1 and TPC2 in mammals) are localized to endo-lysosomal membranes, and have been implicated in the release of lysosomal Ca^2+^ in response to NAADP,^21,27,28^ although their native ionic permeability is controversial.^29,30^

Since the identification of NAADP receptors in the heart over 10 years ago,^31^ a role of NAADP in the cardiac inotropic response has gradually been revealed. Although NAADP does not appear to be involved in baseline cardiomyocyte contraction, there is evidence that it is involved in the increased Ca^2+^ transient and contraction force in response to isoproterenol.^8^ Delivery of NAADP into resting primary cardiomyocytes induces Ca^2+^ release from lysosomes, and evokes slow, cytosolic Ca^2+^ waves reminiscent of those we previously observed in the intact heart during reperfusion.^3,8,32^ Higher concentrations of NAADP also triggered spontaneous diastolic Ca^2+^ waves.^32^ Significantly, administration of an inhibitor of NAADP signalling suppressed diastolic Ca^2+^ waves and also prevented isoproterenol-induced arrhythmias in mice.^32^ Diverse stimuli can increase NAADP within seconds, whereas cardiac NAADP levels were increased 5 min after β-adrenergic stimulation.^8^ Recently, a cell-permeable, small-molecule inhibitor of NAADP signalling was identified in a chemical screen and shown to be effective at nanomolar concentrations *in vitro*.^17^ The compound, Ned-19, eliminated glucose-stimulated Ca^2+^ oscillations in mouse pancreatic beta cells.^17^ Ned-19 similarly inhibited Ca^2+^ oscillations in other tissues such as uterine smooth muscle (stimulated with oxytocin)^24^ and pulmonary arterial myocytes (stimulated with endothelin-1).^18^

As reperfusion-induced Ca^2+^ oscillations lead to mPTP opening and irreversible cell injury, in combination with other factors present at reperfusion, they represent an important potential cardioprotective target. However, the use of canonical Ca^2+^-channel inhibitors in this approach is complicated by the essential role of Ca^2+^ in cardiac contraction. Given the role of NAADP in the generation of Ca^2+^ oscillations in diverse systems including cardiac arrhythmia, we reasoned that NAADP signalling may also play a role in the generation of reperfusion-induced Ca^2+^ oscillations that lead to lethal injury. Using a chemically modified form of Ned-19 called Ned-K, we have demonstrated that inhibition of NAADP-dependent Ca^2+^ oscillations resulted in cardioprotection in both an *in vitro* cell-based assay and an *in vivo* model of ischaemia and reperfusion. Experiments demonstrating that TPC1 knockout mice were similarly protected against ischaemia and reperfusion injury validated the NAADP signalling pathway as a target for cardioprotection. Thus, inhibition of NAADP-stimulated Ca^2+^ oscillations represents a viable cardioprotective strategy which may have minimal effects on regular cardiac contraction.

## 2. Methods

Methods are described in detail in Supplementary material online.

### 2.1 Animal experiments and cardiomyocyte isolation

All animals received humane care in accordance with the United Kingdom Home Office Guide on the Operation of Animal (Scientific Procedures) Act of 1986. The investigation conforms to the guidelines from Directive 2010/63/EU of the European Parliament on the protection of animals used for scientific purposes or the NIH guidelines. Male Sprague–Dawley rats were anaesthetized by i.p. injection of 160 mg/kg pentobarbitone. Male C57BL/6J and TPC1 knockout mice^33,34^ were anaesthetized by i.p. injection (0.01 mL/g) of a solution containing ketamine 10 mg/mL, xylazine 2 mg/mL, and atropine 0.06 mg/mL. Adequacy of anaesthesia was monitored by pedal response and breathing rate. Animals were euthanized by severing of the aorta. The *in vivo* model of myocardial infarction was performed in mice using 30 min ischaemia followed by 120 min reperfusion. Drugs were administered i.v. 5 min before reperfusion. Adult rat ventricular cardiomyocytes were prepared by standard methods.^35^

### 2.2 mPTP assay

mPTP opening was induced in a previously described model of oxidative stress in which cardiomyocytes loaded for 15 min with 5 µM tetra-methyl rhodamine methyl ester (TMRM) are scanned using the 543 nm laser line of a confocal microscope, generating reactive oxygen species (ROS).^35–38^ The time to mitochondrial depolarization provides an index of mPTP sensitivity to opening.

### 2.3 Mitochondrial swelling assay

Mitochondrial swelling was assessed by measuring the absorbance of the mitochondrial suspension (0.5 mg/mL) at 520 nm after the addition of 500 µM free Ca^2+^.

### 2.4 Confocal imaging of Ca^2+^ sparks, transients, and oscillations

To detect Ca^2+^ sparks and transients, cardiomyocytes were loaded with the fluorescent dye Fluo4-AM, 5 µmol/L for 30 min. Rapid line scans were performed (three line scans per cell at different regions of the cell) using the HeNe 488 nm laser of an Leica SP5 confocal microscope. Ca^2+^ spark frequency was determined using ImageJ with the ‘Sparkmaster’ plugin. Ca^2+^ transients were stimulated by electrical field stimulation with platinum electrodes (square pulses, 1 Hz, 1 ms, 5 V/cm). Addition of 10 mmol/L-Caffeine was used to trigger Ca^2+^ release from the SR and measure SR Ca^2+^ content. Cells were subjected to simulated ischaemia by incubating in a glucose-free, anoxic buffer (pH 6.4), before reoxygenation in normoxic buffer. Cell death was determined by staining with propidium iodide (PI).

### 2.5 Chemical synthesis

The trans-form of Ned-19 was synthesized as described previously.^13^ The synthesis of Ned-K is described in Supplementary material online.

### 2.6 Statistics

All values are expressed as mean ± SEM. Data were analysed by one-way ANOVA followed, where significant, by *post hoc* analysis using Dunnett's test for comparisons solely against control values, or Tukey's test for multiple comparisons.

## 3. Results

**Figure 1:**
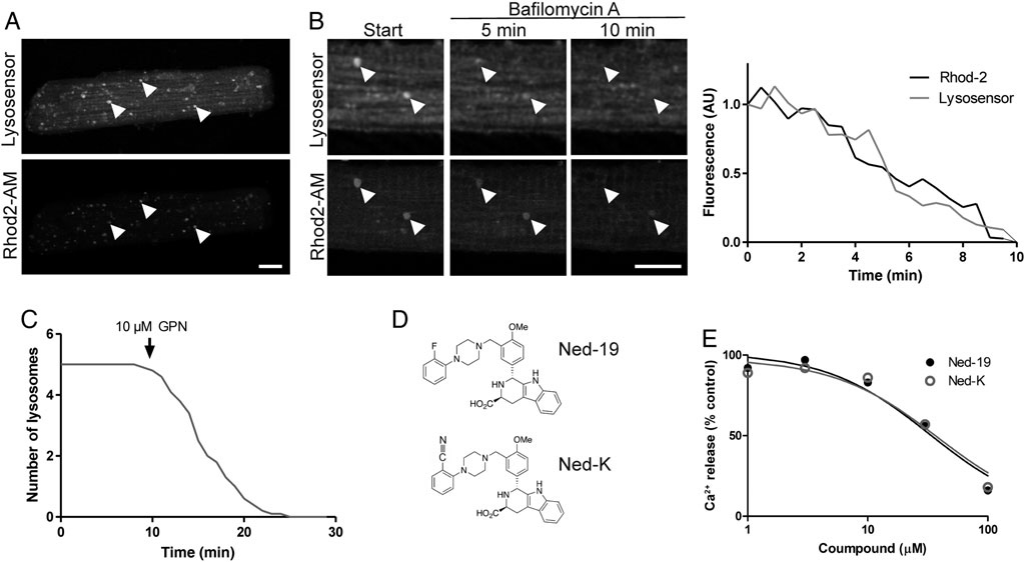
Distribution of lysosomal Ca^2+^ stores in cardiomyocytes and description of Ned-19 and Ned-K. (*A*) Primary rat cardiomyocytes contain abundant lysosomes distributed throughout the cell, many of which contain Ca^2+^ (e.g. arrowheads), as visible in a projected Z-stack of a cardiomyocyte stained with lysosensor green and the Ca^2+^ sensor rhod2-AM. Bar, 10 µm. (*B*) Treatment with 100 nmol/L of bafilomycin A, an inhibitor of vacuolar H^+^-ATPases, caused a gradual loss of both lysosensor and rhod2 fluorescence from lysosomes (arrowheads). Bar, 10 µm. *n* = 3 independent experiments. (*C*) Treatment with 10 µM GPN causes a decrease in the number of lysosomes per cell detected with lysosensor within 10 min. *n* = 3 independent experiments with nine cells. (*D*) The chemical structures of Ned-19 and Ned-K. (*E*) Inhibition curves of Ned-19 and Ned-K in an assay of NAADP-stimulated Ca^2+^ release using sea urchin egg homogenates (see Supplementary material online, Methods). *n* = 3 independent measurements per group.

Lysosomes are Ca^2+^-containing acidic organelles.^39^ Cardiomyocytes contain abundant lysosomes, as detected by staining with the fluorescent lysosomotropic dye lysosensor green (*Figure 1A*). Although rhod2-AM is typically used as a probe of mitochondrial Ca^2+^, it also accumulates in lysosomes as has been previously noted.^40^ There was a high degree of overlap between lysosensor and rhod2-AM fluorescence (*Figure 1A*), indicating that most of the lysosomes in cardiomyocytes contain Ca^2+^. Sequestration of Ca^2+^ in lysosomes depends on their low luminal pH. Consequently, treatment of cells with bafilomycin A, an inhibitor of vacuolar H^+^-ATPases,^41^ resulted in gradual loss of both the pH-dependent, lysosensor probe, and rhod2 fluorescence from the lysosomes (*Figure 1B*). Pretreatment with glycyl-l-phenylalanine-beta-naphthylamide (GPN), which selectively permeabilizes lysosomes by osmotic lysis, also caused gradual disappearance of lysosensor green staining (*Figure 1C*), in parallel with loss of Rhod-2 fluorescence. Interestingly, GPN also caused a transient increase in the frequency of spontaneous Ca^2+^ sparks (see Supplementary material online, *Figure S1*). Thus, lysosomes contain Ca^2+^ and are a potential source of Ca^2+^ release in ventricular cardiomyocytes.

Next, with the aim of improving the biological effectiveness and selectivity of Ned-19, we developed a new analogue of Ned-19, in which the fluoride was replaced with a cyano group (*Figure 1D*). This modification improved its cLogP value, a measure of aqueous solubility calculated by the Cresset Fieldview System, and its topological polar surface area (TPSA), (Ned-19 cLogP = 4.6, TPSA = 80.8; Ned-K cLogP = 4.05, TPSA = 105). Indeed, this new compound, which we called Ned-K, is noticeably more soluble than Ned-19, and it was possible to dissolve it at concentrations up to 300 mmol/L in stock solutions of DMSO, compared with a maximal limit of 100 mmol/L solutions of Ned-19. We compared the potency of Ned-K with Ned-19 using the sea urchin egg homogenate assay system that was used in the original characterization of Ned-19.^17^ Addition of NAADP to this system causes a rapid release of Ca^2+^ from acidic organelles, and compounds such as Ned-19 inhibit this NAADP-stimulated Ca^2+^ release. According to this assay, Ned-K retained an almost identical inhibitory capacity as Ned-19 *in vitro* (*Figure 1E*).

**Figure 2:**
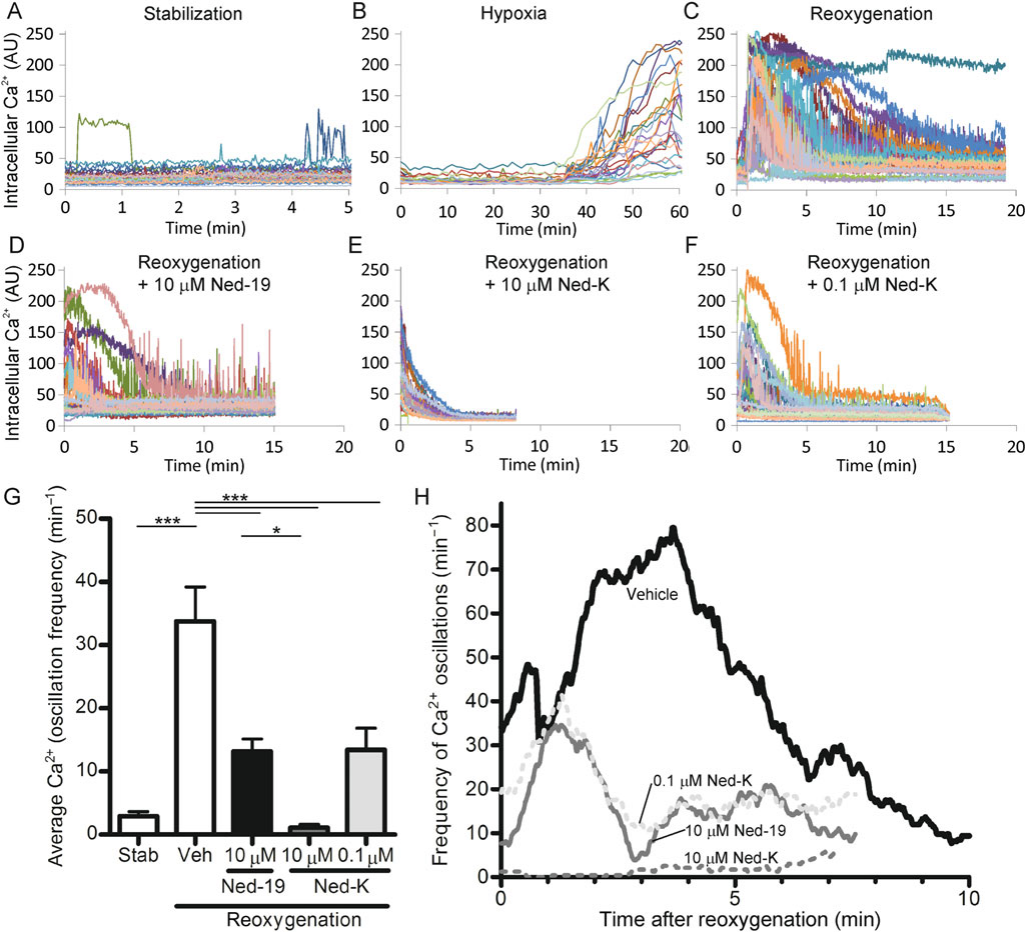
Primary cardiomyocytes subjected to simulated ischaemia and reoxygenation *in vitro* exhibit large Ca^2+^ oscillations at reoxygenation, which are suppressed in the presence of Ned-K or Ned-19. (*A*) Primary cardiomyocytes loaded with a Ca^2+^-sensitive fluorescent indicator, Fluo4-AM, exhibited occasional Ca^2+^ fluctuations during stabilization. (*B*) [Ca^2+^] increased after 40 min hypoxia. (*C*) Large Ca^2+^ oscillations occurred as [Ca^2+^] returned to baseline during reoxygenation. (*D* and *F*) In the presence of 10 µmol/L of Ned-19 (*D*), 10 µmol/L of Ned-K (*E*), or 0.1 µmol/L of Ned-K (*F*), Ca^2+^ oscillations during reoxygenation were suppressed. (*G*) Compared with stabilization, the average number of Ca^2+^ oscillations was significantly increased during reoxygenation, whereas Ca^2+^ oscillations were significantly decreased compared with vehicle by 0.1 µmol/L of Ned-K or 10 µmol/L of Ned-19, and even further suppressed by 10 µmol/L of Ned-K (*P* < 0.05 vs. 0.1 µmol/L of Ned-K; *n* = 3 independent experiments with 26–35 cells per group). (*H*) A smoothed chart of the frequency of Ca^2+^ oscillations that occurred over time during early reoxygenation. ***P < 0.001, *P < 0.05.

Since lysosomal Ca^2+^ triggers ER/SR-based Ca^2+^ oscillations in various cell types, we investigated whether inhibition of NAADP signalling would prevent the Ca^2+^ oscillations which occur at reperfusion and lead to mPTP opening and cell death.^15,42^ First, we examined the changes in [Ca^2+^]_c_ that occur in cardiomyocytes during simulated ischaemia and reperfusion (sIR). Adult rat cardiomyocytes were loaded with the fluorescent Ca^2+^ sensor, Fluo4-AM, and imaged during a period of 60 min simulated ischaemia followed by reoxygenation for 20 min (sIR) on the stage of a confocal microscope. Before ischaemia, [Ca^2+^]_c_ was stable except for occasional spikes during spontaneous cellular contractions, which occur in unstimulated cultures (*Figure 2A*). As expected, [Ca^2+^]_c_ had risen markedly after ∼40 min simulated ischaemia (*Figure 2B*). After reoxygenation, most cells underwent rapid periodic contractions corresponding with large [Ca^2+^]_c_ oscillations (*Figure 2C* and *H*). The frequency of these Ca^2+^ oscillations reached a maximum at ∼3 min, then decreased gradually over the next 7–12 min (*Figure 2C* and *H*), during which time some cells underwent Ca^2+^ overload and hypercontracture (indicating lethal injury), and others regained [Ca^2+^]_c_ homeostasis, recovering normal rod-shaped morphology.

We evaluated the potential of the Ned drugs to inhibit [Ca^2+^]_c_ oscillations during reoxygenation. Ned-19 (10 µmol/L) decreased the frequency of [Ca^2+^]_c_ oscillations (*Figure 2D* and *H*). The chemical modification in Ned-K appears to greatly improve its effectiveness at preventing Ca^2+^ oscillations in this model, since 10 µmol/L of Ned-K almost completely eliminated [Ca^2+^]_c_ oscillations (*Figure 2E* and *H*), and 0.1 µmol/L of Ned-K was as effective at suppressing [Ca^2+^]_c_ levels as 10 µmol/L of Ned-19 (*Figure 2D*, *F*, and *H*). The average frequency of Ca^2+^ oscillations in each cell was analysed statistically (see Methods; *Figure 2G*) and was found to be significantly suppressed by the treatments (*P* < 0.001, *n* = 3). These results demonstrate that Ned-19 and, particularly, Ned-K were effective at dampening sIR-induced Ca^2+^ oscillations in cardiomyocytes.

**Figure 3:**
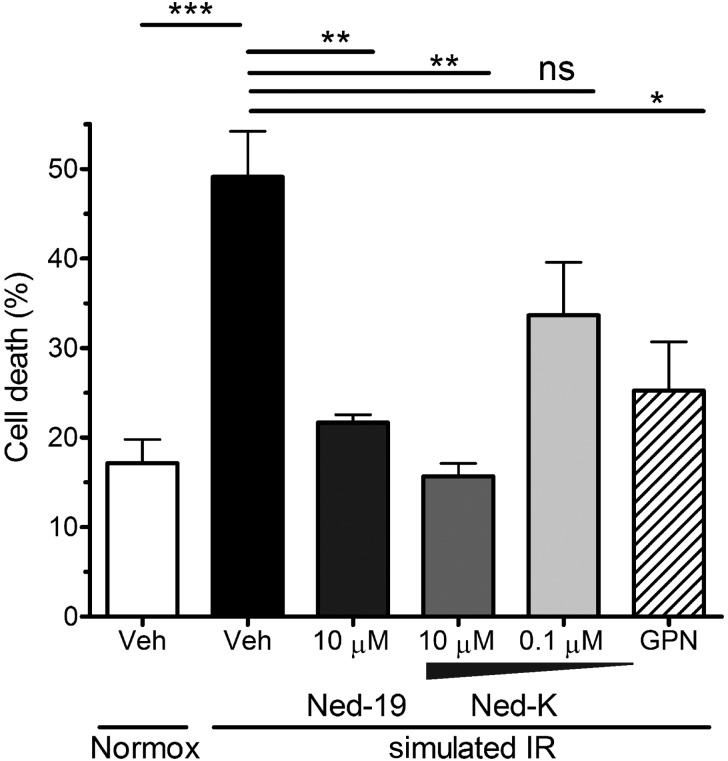
sIR increases the percentage of dead primary adult cardiomyocytes (i.e. hypercontracted and PI+ve). Treatment with 10 µmol/L of Ned-K or 10 µmol/L of Ned-19 significantly decreased the percentage of dead cells. Pretreatment of cells with 10 µmol/L of GPN, which eliminates lysosomes, was also protective. *P < 0.05; **P < 0.01; ***P < 0.001; *n* = 4 independent experiments with 700–1500 cells per group.

To determine whether inhibition of NAADP-stimulated Ca^2+^ oscillations corresponded to protection against ischaemia and reperfusion injury, the cells were stained with vital dye PI at the end of the experiment, and the percentage of dead vs. live, rod-shaped cells was scored. sIR significantly increased the percentage of dead cells to 49 ± 5% compared with 17 ± 3% in normoxic buffer (*P* < 0.001, *n* = 4; *Figure 3*). Treatment with 10 µmol/L of Ned-19 or 10 μmol/L of Ned-K at reoxygenation significantly decreased cell death after sIR to 22 ± 1 and 16 ± 1%, respectively (both *P* < 0.01 vs. vehicle). Ned-K (0.1 µmol/L) caused a slight decrease in cardiomyocyte death (34 ± 6%), which did not reach significance. We reasoned that if protection occurs via inhibition of lysosomal Ca^2+^ release, then an agent that eliminates lysosomal Ca^2+^ would be similarly protective. Indeed, pretreatment of cells with GPN to permeabilize lysosomes, followed by 30 min washout before sIR, decreased cell death to 25 ± 5% (*P* < 0.05 vs. vehicle). Brief pretreatment of cells with GPN also suppressed [Ca^2+^]_c_ oscillations at reperfusion to a similar degree (see Supplementary material online, *Figure S2A–D*).

**Figure 4:**
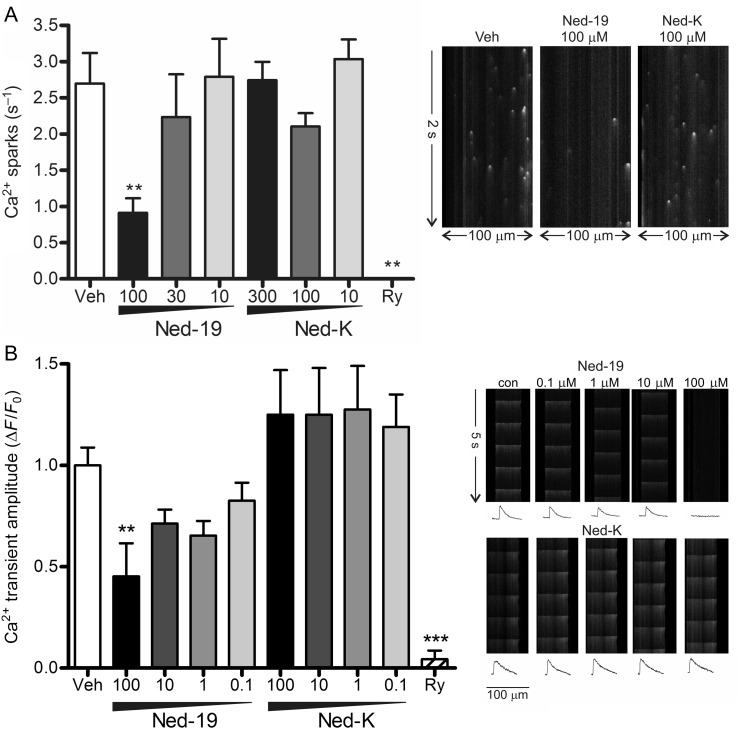
Ned-19 but not Ned-K adversely affected spontaneous SR Ca^2+^ sparks and pacing-induced Ca^2+^ transients in confocal line scans of cardiomyocytes loaded with fluo4-AM. (*A*) The frequency of Ca^2+^ sparks in the presence of Ned-19 or Ned-K (*n* = 14–16 cells). Ryanodine (Ry, 10 µmol/L) eliminated Ca^2+^ sparks (*n* = 3 independent experiments with 10–30 cells per group). Representative line scans are shown. **P < 0.01 vs. vehicle. (*B*) The amplitude of electrically stimulated Ca^2+^ transients (Δ*F*/*F*_0_) was significantly decreased by 100 µmol/L of Ned-19. In contrast, Ned-K had no effect. Representative line scans are shown, with traces of Ca^2+^ transients indicated below. **P < 0.01, ***P < 0.001; *n* = 6 independent experiments with 30–40 cells per group.

To confirm that the protection and suppression of Ca^2+^ oscillations observed with Ned-19 and Ned-K was not due to off-target inhibition of the RyR directly, we evaluated their effect on the frequency of spontaneous Ca^2+^ sparks, and on efficiency of excitation–contraction coupling. Ca^2+^ sparks occur spontaneously in unstimulated primary cardiomyocytes primarily as a consequence of spontaneous Ca^2+^ release from the SR via the RyR,^43^ as demonstrated by the complete inhibition of Ca^2+^ sparks by ryanodine (Ry; *Figure 4A*). The frequency of Ca^2+^ sparks in cardiomyocytes treated with different concentrations of Ned-K was not significantly altered, even at concentrations up to 300 µmol/L (*Figure 4A*). However, at the highest concentration used (100 µmol/L), Ned-19 inhibited Ca^2+^ spark frequency from 2.7 ± 0.4 Ca^2+^ sparks/s in controls to 0.9 ± 0.2 Ca^2+^ sparks/s (*P* < 0.01, *n* = 3; *Figure 4A*). Similarly, while Ca^2+^ transient amplitude in response to electrical pacing was unaltered by the presence of 100 µmol/L of Ned-K (*Figure 4B*), their amplitude was significantly decreased by 100 µmol/L of Ned-19 (*Figure 4B*), with Ca^2+^ transients completely eliminated in 30% of cells analysed. These results suggested that 100 µmol/L of Ned-19 affects SR Ca^2+^ handling either by inhibiting the RyR or decreasing SR Ca^2+^ content, and subsequent experiments measuring the release of SR Ca^2+^ by treatment with caffeine indicated that the latter is the case (see Supplementary material online, *Figure S3*). Ned-K, however, appears to be more specific as it has no such direct effect on SR Ca^2+^ handling.

**Figure 5:**
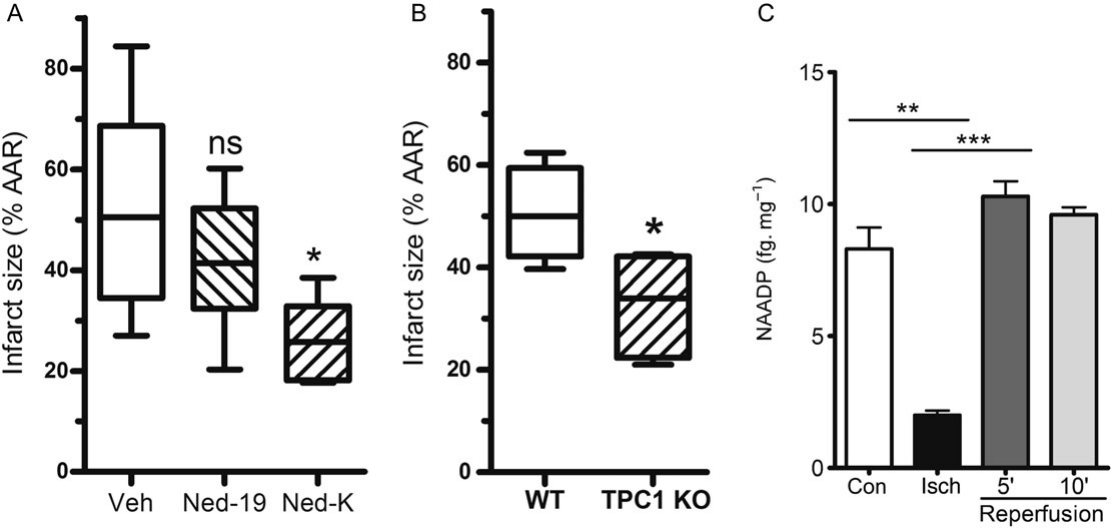
Evaluation of cardioprotection in an *in vivo* model of ischaemia and reperfusion. (*A*) Ned-K, but not Ned-19 (administered i.v. to mice 5 min before reperfusion), significantly decreased myocardial infarct size relative to area at risk. *P < 0.05; *n* = 5–6 per group. (*B*) Infarct size relative to area at risk was significantly decreased in mice lacking TPC1. See Supplementary material online, *Figure S4* for representative heart sections after tetrazolium staining. *P < 0.05; *n* = 4–5 per group. (*C*) NAADP levels in hearts subject to 30 min normal perfusion (con), ischaemia (Isch), or ischaemia followed by reperfusion for 5 or 10 min. **P < 0.01, ***P < 0.001; *n* = 3 hearts per group.

Next, we investigated whether Ned-19 or Ned-K would protect against cardiac ischaemia and reperfusion injury in an *in vivo* mouse model of ischaemia and reperfusion injury. The left anterior descending coronary artery of anaesthetized mice was occluded for 30 min, followed by a period of reperfusion for 2 h, after which time the heart was removed and the extent of infarction in the area at risk measured by tetrazolium chloride staining and Evans blue. In control mice, injected with the vehicle 5 min before reperfusion, infarct size measured 51 ± 9% (*n* = 5) of the ischaemic area ‘at risk’ (*Figure 5A* and see Supplementary material online, *Figure S4*). The infarct size in mice injected with Ned-19 was slightly lower but not significantly different from controls (41 ± 5%, *n* = 6; *Figure 5A*). Injection of Ned-K, however, caused a significant reduction in infarct size (25 ± 3%, *n* = 5; *P* < 0.05; *Figure 5A*).

We tested whether genetic suppression of the NAADP signalling pathway would be similarly cardioprotective. TPC1 has been proposed to form a Ca^2+^ channel in the lysosomal membrane that is regulated by NAADP.^21,27^ We therefore probed the role of TPC1 in injury using mice lacking TPC1.^33^ TPC1 knockout mice have no obvious phenotype, but after subjecting them to experimental myocardial infarction they were revealed to be protected against cardiac ischaemia and reperfusion, with infarct sizes of 32 ± 4% of the area at risk, compared with 51 ± 5% of the area at risk in littermate wild-type controls (*n* = 4–5, *P* < 0.05; *Figure 5B*). Taken together, the data in *Figure 5A* and *B* show that both chemical and molecular inhibition of NAADP signalling *in vivo* reduces ischaemia and reperfusion injury.

To test whether ischaemia and reperfusion injury was associated with changes in NAADP levels, mouse hearts were isolated and perfused using a Langendorff apparatus. An improved enzymatic cycling assay^44^ was used to quantify NAADP in hearts subjected to global ischaemia and reperfusion. NAADP levels in control perfused mouse hearts were 8.3 ± 2.0 fmol mg^−1^ protein (*Figure 5C*). NAADP was significantly reduced after a period of 30 min global ischaemia to 2.0 ± 0.3 fmol mg^−1^. Upon reperfusion, NAADP levels recovered, though they did not increase above baseline levels. However, these data show that NAADP levels are dynamically regulated during ischaemia and reperfusion further supporting a role for NAADP signalling in injury at reperfusion.

**Figure 6:**
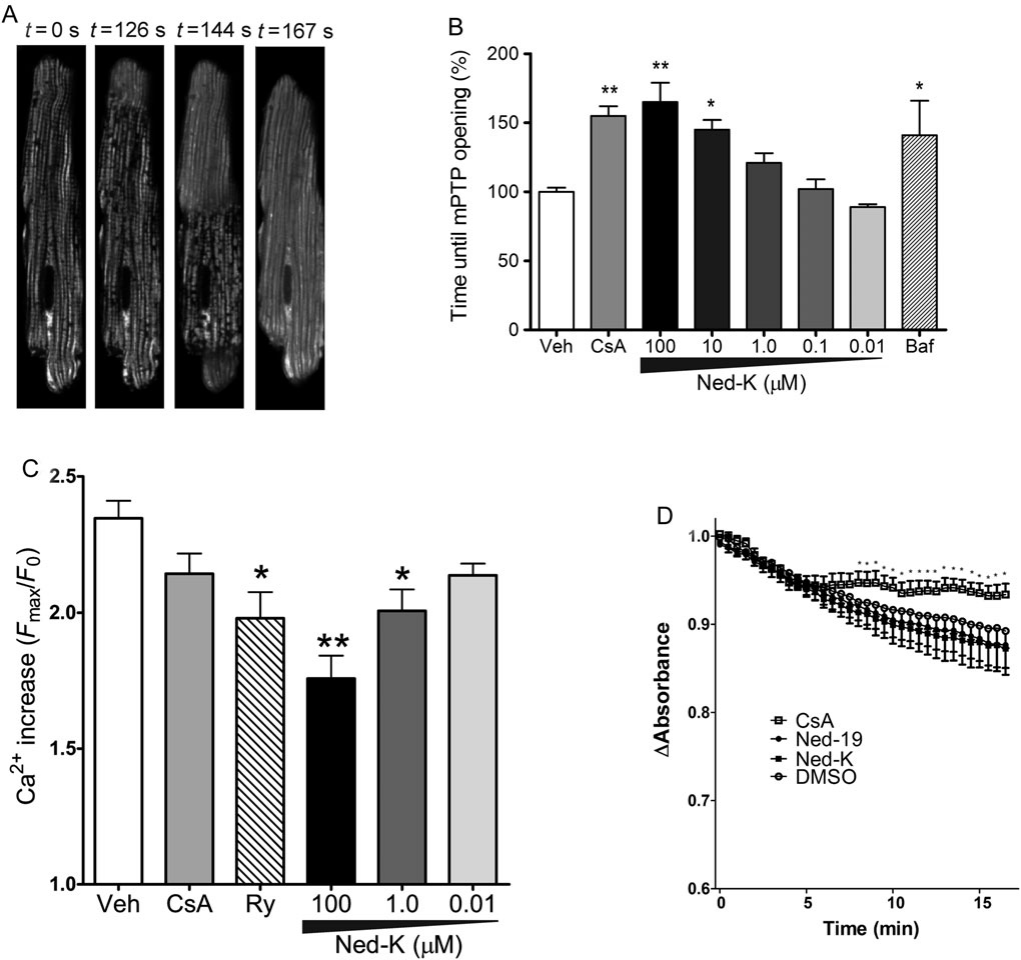
Ned-K increased the resistance of the mPTP to opening in response to oxidative stress. (*A*) Confocal images demonstrating the model in which mPTP opening leads to a de-quench wave of fluorescence across the cardiomyocyte. (*B*) The time to mPTP opening was assessed in cells exposed to oxidative stress after treatment with the indicated drugs. Ned-K delayed mPTP opening, as did CsA, an inhibitor of the mPTP. Emptying of acidic stores with 100 nmol/L of bafilomycin A (Baf) also delayed mPTP opening. *n* = 4 independent experiments with 18–46 cells per group. *P < 0.05, **P < 0.01, vs. vehicle control. (*C*) Ca^2+^ increased during the mPTP assay, and this was blunted by 10 µmol/L of ryanodine (‘Ry’), or Ned-K (*n* = 4 independent experiments). (*D*) Ned-K (10 µmol/L) did not directly affect the Ca^2+^ threshold for mPTP opening in isolated mitochondria when compared with the mPTP inhibitor, 0.2 µmol/L of CsA. *n* = 6 independent experiments.

Finally, we investigated the mechanism of cardioprotection by Ned-K by examining whether it could prevent opening of the mPTP. We used a previously validated model,^45,46^ in which primary adult cardiomyocytes are loaded with the fluorescent dye TMRM, which accumulates in mitochondria and generates ROS in response to confocal laser-induced phototoxicity. This results in Ca^2+^ overload culminating in mPTP opening and cell death.^46^ We measured the time taken under continual confocal laser scanning before mPTP opening occurs, visualized by the rapid redistribution of TMRM from the mitochondria to the cytosol, and by an abrupt increase in fluorescence as the dye dequenches in the cytosol (*Figure 6A*). As a positive control, ciclosporin A (CsA) significantly delayed the time to mPTP opening compared with vehicle by 55 ± 7% (*n* = 4, *P* < 0.01; *Figure 6B*). There was a significant delay in mPTP opening in the presence of 10 or 100 µmol/L of Ned-K (*Figure 6B*) or Ned-19 (see Supplementary material online, *Figure S5*). In this model, ROS exposure caused a 2.4-fold increase in [Ca^2+^]_cyto_, detected using Fluo4-AM (see Supplementary material online, *Figure S6*), and this increase was suppressed in the presence of 10 µmol/L of ryanodine or, 1–100 µmol/L of Ned-K (*Figure 6C*). Since this is the first time that a link between Ca^2+^ release from acidic stores and mPTP opening has been demonstrated, we performed a further experiment in which cardiomyocytes were pretreated for 30 min with bafilomycin A to assess whether this would have any effect on mPTP opening. In line with our hypothesis, pretreatment with 100 nM bafilomycin A significantly delayed the time to mPTP opening by 41 ± 25% (*P* < 0.05, *n* = 4; *Figure 6B*). The delay in mPTP opening was not due to a direct effect of the Ned drugs on the mPTP, since Ca^2+^-induced swelling of isolated mitochondria was unaffected by their presence (*Figure 6D*).

## 4. Discussion

Our results demonstrate that reperfusion-induced Ca^2+^ oscillations in primary adult cardiomyocytes can be suppressed by treatment with Ned-19, or, more effectively, with an improved form of Ned-19 called Ned-K. Although Ned-19 decreased SR Ca^2+^ content at higher concentrations, Ned-K had no effect on SR Ca^2+^ content, and did not alter spontaneous Ca^2+^ spark frequency or electrically stimulated Ca^2+^ transient amplitude. While both drugs protected primary cardiomyocytes against sIR injury *in vitro*, Ned-K was uniquely protective against IR injury *in vivo*, in accordance with its improved pharmacological properties. Genetic deletion of TPC1 was similarly protective in mice. The protective effects of Ned-K were mirrored *in vitro* by treatment with GPN to eliminate lysosomes. Mechanistically, protection is likely via prevention of reperfusion-induced Ca^2+^ oscillations that trigger mPTP opening in cardiomyocytes.

The mechanism of action of NAADP is not entirely clear. Increasing evidence supports a ‘trigger mechanism’ in which NAADP causes release of Ca^2+^ from lysosomes which then triggers SR/ER Ca^2+^ release,^19,47–49^ in much the same way as sarcolemmal Ca^2+^ entry triggers SR Ca^2+^ release during excitation–contraction coupling.^47^ Indeed, in recent years, lysosomes have been gaining attention, with the growing realization that their function extends beyond the ‘garbage disposal’ role in protein degradation originally ascribed to them. They are now recognized as an important store of Ca^2+^, containing ∼400–600 µmol/L in macrophages, and ∼550 µmol/L in fibroblasts.^25,39^ Our observation that pretreatment of cardiomyocytes with bafilomycin A to deplete lysosomal Ca^2+^, both delayed mPTP opening and protected against sIR, supports a mechanism in which NAADP stimulates the release of lysosomal Ca^2+^, which then triggers Ca^2+^ oscillations during reperfusion.

Interestingly, there was a significant reduction in total cardiac NAADP content after a period of 30 min global ischaemia which returned to basal levels by 5 min reperfusion. We speculate that oxidation of the lysosomal Ca^2+^ channel during reperfusion may sensitize it to NAADP, in a manner similar to the redox regulation of RyR2.^50^ We have previously shown that intracellular oxidation and cytosolic Ca^2+^ overload occur together in highly localized regions during reperfusion of the myocardium, leading to mPTP opening.^3^

Reperfusion-induced Ca^2+^ oscillations are caused by the cyclic release and uptake of Ca^2+^ from the SR. While we have shown that inhibition of lysosomal release channels reduces these oscillations, other mechanisms could affect Ca^2+^ SR load and triggering of SR Ca^2+^ release during ischaemia or reperfusion, such as ATP depletion which prevents Ca^2+^ pumps from functioning. Other transporters, such as the plasmalemmal Na^+^/Ca^2+^ exchanger, contribute indirectly to Ca^2+^ oscillations by increasing cytosolic Ca^2+^ overload during reperfusion.^51,52^ Notably, in these studies, the complete absence of extracellular Ca^2+^ during reperfusion only reduced the oscillation frequency by ∼50%,^51^ which reflects the fact that internal Ca^2+^ sources must contribute to the oscillations.

Some differences were observed between Ned-19 and Ned-K, which may be ascribed to the changes in chemical structure. Ned-19 was previously identified in a virtual screen using NAADP as the query ligand. Using the sea urchin egg homogenate assay, it was verified as inhibiting NAADP-stimulated Ca^2+^ release activity. Similarly, Ned-19 inhibited NAADP-induced Ca^2+^ release in mouse pancreatic beta cells, and reduced glucose-induced Ca^2+^ increase in islets. By replacing the fluoride in Ned-19 with a cyano group in Ned-K (*Figure 1*), we improved its cLogP value from 4.6 to 4.05. Although the change in cLogP value may appear marginal, this is a tangible outcome given that values are logarithmic. The TPSA is a measure of the polar contribution of groups such as O, N, and –CN and is related to how hydrated a molecule can become in aqueous environments. This value was improved from 80.8 in Ned-19 to 105 in Ned-K. More importantly, it seems that the receptor sites or ion channels involved in cardioprotection may have water molecules that can bridge between the peptide residues and the cyano group in Ned-K.

Opening of the mPTP is a major step on the path to cell necrosis and IR injury, as shown by experiments with mice lacking the cypD component of the mPTP or treated with CsA, which binds to and inhibits cypD.^1,2,53^ Given that NAADP stimulates Ca^2+^ oscillations, and Ca^2+^ oscillations cause mitochondrial Ca^2+^ overload and mPTP opening, we hypothesized that the mechanism of protection of NAADP inhibitors would be via inhibition of the mPTP. We used a model of ROS-induced mPTP opening in primary cardiomyocytes. Ca^2+^ was shown to increase in this model and contribute to mPTP opening. The involvement of lysosomes in this model was confirmed by the delay in mPTP opening observed after pretreatment with bafilomycin A to empty acidic stores (*Figure 6B*). In accordance with our hypothesis, we found that Ned-19 and Ned-K inhibited mPTP opening in cells (*Figure 6B*), but that this effect was indirect, since they did not prevent Ca^2+^-induced swelling in isolated mitochondria (*Figure 6D*). The mPTP represents a common end target of many pharmacological cardioprotective agents, although often the exact mechanism by which they prevent mPTP opening is not known.^35,54^ It may be interesting to evaluate whether any of these agents affect lysosomal Ca^2+^ release.

Despite Ned-19 and Ned-K having similar activities in an *in vitro*, sea urchin egg homogenate assay of NAADP inhibition, only Ned-K was found to be cardioprotective *in vivo*. The exact reason for this difference is not known, but may be related to differences in pharmacokinetic profile. An alternative possibility is that Ned-19 has an additional detrimental off-target effect—perhaps one related to the effect it was observed to have *in vitro* on depleting SR Ca^2+^. We attempted to measure tissue content of Ned compounds by HPLC after administration *in vivo*, but the levels were at or below the lower limit of detection. A further limitation of the study is that we did not test cardioprotection of Ned-K in the TPC1 knockout mice, which would have helped to confirm its mechanism of action.

Deletion of TPC1 protected against ischaemia and reperfusion injury, suggesting that it is functionally important in the ventricular myocardium. Much evidence has accumulated recently identifying TPCs as NAADP-regulated Ca^2+^-permeable channels.^8,16–22,28^ NAADP-mediated Ca^2+^ signals are thus potentiated when TPCs are overexpressed and inhibited by siRNA, gene knockout, and dominant-negative TPC constructs (reviewed in Hooper and Patel^48^). In two thorough, but highly controversial publications, data have been presented indicating that TPCs are not Ca^2+^ channels, but rather Na^+^-selective ion channels that are insensitive to NAADP and instead activated by the phosphoinositide PI(3,5)P_2_ and ATP depletion.^29,55^ These discrepancies have been partly reconciled by a recent study which defined ionic conditions uncovering co-regulation of TPCs by NAADP, PI(3,5)P_2_, and ATP.^56^ Thus, during ischaemia, ATP depletion might contribute to activation of TPCs by NAADP and/or PI(3,5)P_2_. Further work, however, is required to determine whether NAADP-dependent Ca^2+^ oscillations reported here are a direct consequence of Ca^2+^ flux through TPCs. Nevertheless, recent pharmacological^57^ and genetic^58,59^ approaches reaffirm the importance of TPCs in NAADP-mediated Ca^2+^ release.^28^ In support, both NAADP antagonism and TPC1 knockout afford protection against reperfusion injury highlighting the therapeutic potential of this pathway.

## Supplementary material

Supplementary material is available at *Cardiovascular Research* online.

## Funding

This work was supported by MRC (EAA/17568), the British Heart Foundation (PG-10-005), and by the Biotechnology and Biological Sciences Research Council grant numbers (BB/G013721/1 and BB/K000942/1). The work was undertaken at UCLH/UCL who received a proportion of funding from the Department of Health's NIHR Biomedical Research Centres funding scheme. Funding to pay the Open Access publication charges for this article was provided by the MRC and BHF.
